# Pharmacological Activities and Synthesis of Esculetin and Its Derivatives: A Mini-Review

**DOI:** 10.3390/molecules22030387

**Published:** 2017-03-02

**Authors:** Chengyuan Liang, Weihui Ju, Shaomeng Pei, Yonghong Tang, Yadong Xiao

**Affiliations:** 1 Faculty of Pharmacy, Shaanxi University of Science & Technology, 6 Xuefu Road, Xi’an 710021, China; chengyuanliang@gmail.com (C.L.); juweihui11521@sina.com (W.J.); huabienaiqiao@sina.com (S.P.); 2 Xi’an Taikomed Pharmaceutical technology Co., LTD, Xi’an 710077, China; techno8833@taikomed.com; 3 Faculty of Finance and Economics, Guangzhou Vocational College of Science and Technology, Guangzhou 510550, China

**Keywords:** esculetin, coumarin, synthetic derivatives, pharmacological activity, molecular mechanism

## Abstract

Esculetin, synonymous with 6,7-dihydroxycoumarin, is the main active ingredient of the traditional Chinese medicine *Cortex Fraxini*. The twig skin or trunk bark of *Cortex Fraxini* are used by herb doctors as a mild, bitter liver and gallbladder meridians’ nontoxic drug as well as dietary supplement. Recently, with a variety of novel esculetin derivatives being reported, the molecular mechanism research as well as clinical application of *Cortex Fraxini* and esculetin are becoming more attractive. This mini-review will consolidate what is known about the biological activities, the mechanism of esculetin and its synthetic derivatives over the past decade in addition to providing a brief synopsis of the properties of esculetin.

## 1. Introduction

**Figure 1:**
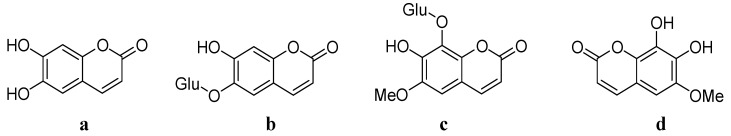
Structures of the contents of *Cortex Fraxini.*

Chinese herbal medicine has been widely used for centuries for the treatment of different diseases. *Cortex Fraxini* is one of the commonly used traditional Chinese medicines (TCMs). According to the TCM division and classification, there are four classified species of *Cortex Fraxini*: *Fraxinus rhynchop hylla Hance*, F. *chinensis Roxb.*, F. *aboana Lingelsh.* and F. *stylosa Lingelsh.* It has been indicated that *Cortex Fraxini* possesses various pharmacological effects, including anti-pathogenic [1], anti-inflammatory [2], analgesic [3], anti-cancer [4], antioxidative stress [5], neuroprotective [6] and vascular protective effects [7]. There are many active ingredients in *Cortex Fraxini*. Among all of the contents of *Cortex Fraxini* (Figure 1), esculetin **a**, esculin **b**, fraxin **c** and fraxetin **d** have been investigated as major pharmacologically active ingredients.

As one of the main active ingredients of *Cortex Fraxini*, esculetin has been widely used not only in expectorant, antitussive aspects [8] but also as anti-inflammatory, antioxidant, antibacterial [9], and antitumour therapeutics. According to its multiple pharmacological effects and modifiable structure, esculetin has been a promising lead for medicinal chemists developing novel drug candidates. The 6,7-biphenolic hydroxyl and 3,4-unsaturated bond of the structure are the vital reactive sites for synthetic modification, which result in novel esculetin derivatives for pharmacological screening. This review covers recent studies on the synthesis, pharmacological activities and mechanism of esculetin and its derivatives over the past decade.

## 2. Pharmacological Effects of Esculetin

### 2.1. Anti-Inflammatory Effects

It was revealed by several reports of molecular mechanisms that esculetin shows activity as an anti-inflammatory agent. On the one hand, esculetin reduces the secretion of NO to regulate blood vessels and eases the organ tissue damage from inflammation; on the other hand, esculetin inhibits the secretion of soluble intercellular adhesion molecule (sICAM-1), which can reduce the adhesion reaction of leukocytes as well as endothelial cells in order to reduce inflammation [10]. Additionally, esculetin can significantly reduce the expression of Matrix metalloproteinase-1 (MMP-1) in cartilage, lower NO and Prostaglandin E2 (PGE2) levels in synovial fluid, and postpone the occurrence of osteo-arthritis [11]. In another experiment, esculetin was found to protect myocardial from ischaemia reperfusion injury by reducing systemic inflammatory responses [12]. Esculetin also exhibits anti-inflammatory properties by inhibiting the production of pro-inflammatory cytokines during the interaction between adipocytes and macrophages, which can be recorded by haeme oxygenase-1 (HO-1) expression. As is well-known, obesity is closely related to chronic low-grade inflammation of adipose tissue. Esculetin may have the potential to slow down chronic inflammation in obesity [13].

### 2.2. Anticoagulant

In view of natural coumarin derivatives such as esculetin, fraxetin, and warfarin and their clinical use as anticoagulant agents [14], esculetin may also have medicinal potential for the treatment of stroke. The proliferation of vascular smooth muscle cells (VSMCs) induced by injury to the intima of arteries is one of the vital pathogenic factors giving rise to vascular proliferative disorders, such as atherosclerosis and restenosis. Esculetin can effectively inhibit the proliferation of VSMCs in vitro in both dose- and time- dependent manners. The mechanism of the antiproliferative effect was revealed by esculetin inhibiting the activation of Ras-Raf-MEK-ERK/MAPK and Ras-PI3K-Akt signalling pathways [15]. Specifically, esculetin blocks’ cell proliferation via mediation of both upstream influence and downstream events of Ras signalling, such as p42/44 MAPK activation, PI3K activation, and early gene expression, as well as NF-kappaB and AP-1 activation. Esculetin also prevents intimal hyperplasia in rat balloon vascular injury models, indicating the curative potential of esculetin for treating restenosis after arterial injury [16]. Another experiment revealed that esculetin can activate nuclear hormone receptor PPAR-γ and promote ABCA1 and ABCG1 expression; then, it inhibits the formation of smooth muscle-derived foam cells [17].

Furthermore, esculetin exerts anti-apoptotic activity on murine brain microvascular endothelial cells by upregulating the expression of Bcl-2, downregulating the expression of Bax and downstream cleaving caspase 3; as a result, esculetin has neuro-protective effects on cerebral ischaemia/reperfusion (I/R) injury in the mouse middle cerebral artery occlusion model [18].

### 2.3. Antioxidant

Free radicals and reactive oxygen species (ROS), which are chemical radicals and reactive species containing oxygen produced naturally in vivo, are natural by-products of the normal metabolism of oxygen and are a result of disease processes or through xenobiotic activities. From a biological perspective, normal ROS levels have important roles in cell signalling and homeostasis. However, dramatically increased ROS levels may result in significant damage to cell structures [19]. Cumulatively, this is known as oxidative stress. As reported, esculetin illustrates the strong scavenging activity against DPPH radicals, the ability of scavenging free radicals shows significant linear correlation with esculetin in both time- and concentration-dependent manners [20]. Esculetin is also a potent agent in protecting cells from ROS-mediated Abeta-damage [21]. In another study, esculetin is effective in protecting cells against DNA damage induced by oxidative stress [22].

### 2.4. Liver-Protective Effects

In liver diseases, increasing ROS and peroxidation of lipids can enhance the production of the extracellular matrix, which is characterized by active proliferation of hepatic stellate cells and accelerated damage to hepatic tissue [23]. A short-chain analogue of lipid hydroperoxide, *t*-butyl hydroperoxide (*t*-BHP), can be metabolized to free radical intermediates by cytochrome P-450 in hepatocytes, which can conversely initiate lipid peroxidation, affect cell integrity and lead to cell damage [24]. Parenchymal-nonparenchymal cross-talk cells are one of the key issues in liver injury, of which ROS plays an important role in increasing platelet-derived growth factor throughout liver fibrogenesis. The traditional Chinese medicines *Cichorium intybus* [25] and *Bougainvllra spectabillis* containing esculetin were endowed with folkloric applications in treating liver damage over a long period. This long-term utilization is positive and proves that esculetin possesses anti-hepatotoxic activity from the perspective of animal studies [26]. Histopathological evaluation revealed that esculetin reduced oxidative stress in a *t*-BHP induced a rat liver lesion model, which could be characterized by decreased liver cell swelling, reduced leukocyte infiltration and necrosis [27]. Thus, esculetin may play a preventative role that through joining utilization with radical-generating agents to release hepatotoxicity.

### 2.5. Antidiabetic Effects

Diabetes mellitus is one of the most common metabolic disorder diseases and it is considered to be one of the leading causes of death worldwide. Hyperglycaemia-mediated oxidative stress plays a crucial role in diabetic complications. Based on the research of Prabakaran, esculetin treatment exerts a protective effect in diabetes by attenuating hyperglycaemia-mediated oxidative stress via antioxidant competence in both hepatic and renal tissues [28]. In another experiment, esculetin exerts a pronounced anti-hyperglycemic effect against streptozotocin-induced diabetic rats [29].

### 2.6. Antibacterial Effects

The human pathogen *Escherichia coli* O157:H7 is one of the Shiga toxin-producing types of *E. coli*, which is thought to be spread by direct or indirect contact with infected animal or human faeces. *E. coli* O157:H7 is the most common pathogen of haemorrhagic colitis, and no effective therapy has been discovered for *E. coli* O157:H7 infection until now. Duncan investigated the effects of the aglycone esculetin on the survival of experimentally infected *E. coli* O157 under gut conditions. The pathogen was detected to be significantly decreased in experimental post-inoculation faecal samples compared with control calves not fed esculin (18% versus 37%). The addition of esculetin to human faecal slurries and in vitro continuous-flow fermenter models simulated conditions in the human colon and rumen, causing marked decreases in the survival of the introduced strain *E. coli* O157 [30]. Another experiment proved that esculetin can repress the Shiga-like toxin gene stx2 in *E. coli* O157:H7 and weaken its virulence in vivo in the nematode *Caenorhabditis elegans* [31].

### 2.7. Antitumour Effects

Phenolic esculetin is found to selectively induce tumour apoptosis in several types of human cancer cells, such as oral squamous cell carcinoma (OSCC) cell lines, HN22, HSC4, human monocytic leukaemia U937 cells and human malignant melanoma (HMM) cells G361 [32]. In a mechanistic study, esculetin was identified as potential Wnt-β-catenin pathway inhibitor by disrupting the formation of the β-catenin-Tcf complex while binding directly with the Lys345, Asn387, Lys312, and Gly307 residues of β-catenin. In addition, esculetin effectively antagonized the formation of the β-catenin-Tcf complex, which could contribute to β-catenin-dependent activity and suppress colon carcinogenesis [33]. Another study investigated the anti-proliferative effects of esculetin on tumour necrosis factor (TNF)-related apoptosis-inducing ligand (TRAIL) induced apoptosis primarily through upregulation of apoptotic related DR5 proteins and correlative immunocytochemistry. Interestingly, esculetin also had a significant anti-proliferative effect and induced apoptosis by suppressing Sp1 transcription factor (Sp1) and modulated downstream target regulatory genes including p27, p21 and cyclin D1 on HN22, HSC4 and HMM cells in time- and dose-dependent manners. Furthermore, esculetin resulted in the activation of apoptosis signalling molecules such as the ERK pathway, caspase-3 and PARP in G361 HMM cells [34]. Notably, all of the abovementioned experiments suggested that esculetin be considered as a promising chemotherapeutic agent candidate in the treatment of acute promyelocytic leukaemia, malignant melanoma and other carcinomas.

### 2.8. Suppressed Adipogenesis

The adipose tissue derived from preadipocytes has been traditionally known as a passive source of energy stores and body heat generation. Loose connective fat tissue is known as a very active endocrine organ producing endogenous active substances such as resistin, estrogen, leptin, and the cytokine TNFα. However, excessive amounts of adipose tissue, specifically in overweight and obesity subjects, is one of the metabolic syndrome risk factors that predisposes the subject to a large number of inflammatory related diseases. Esculetin has the effect of promoting glucose metabolism and mediates adipocyte apoptosis by the mitochondrial pathway initiating the apoptotic process of 3T3-L1 adipocytes [35]. Another experiment indicated that esculetin has anti-adipogenic effects through modulation of PPAR-γ and C/EBPα via the AMPK signalling pathway [36].

## 3. Synthesis of Esculetin

**Scheme 1:**
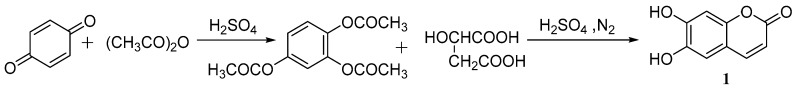
Synthesis route of esculetin.

Cao et al. [37] studied the synthesis of esculetin. *p*-Benzoquinone, acetic anhydride and sulfuric acid were used as the raw materials to produce a 1,2,4-phloroglucinol triacetate intermediate and then combined with concentrated sulfuric acid and malic acid to synthesize aesculetin. The total yield was 80.3% (Scheme 1).

**Scheme 2:**
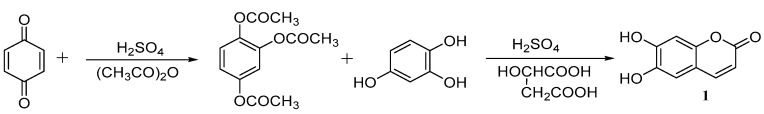
Synthesis of esculetin.

Zhang [38] designed another synthetic route and optimized the reaction conditions by single- and multi-factor orthogonal experiments. The optimal reaction conditions are a ratio of *p*-benzoquinone: acetoacetate: concentrated sulfuric acid of 1:3:0.15 at 45 °C and stirred for 3 h; then, the intermediate peracetylated 1,2,4-benzenetriol is fused with equivalent amount of l,2,4-benzenetriol catalysed by concentrated sulfuric acid and malic acid, and the yield of esculetin can reach approximately 80% (Scheme 2).

**Scheme 3:**
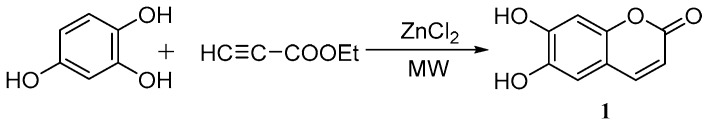
Microwave irradiation synthesis of esculetin.

Yang et al. [39] used the cyclization of 1,2,4-benzenetriol and ethyl propionate to synthesize esculetin under microwave irradiation using ZnCl_2_ as a catalyst. The optimum reaction conditions were as follows: *n*(1,2,4-benzenetriol):*n*(ethyl propionate) = 1.0:1.0, with 3.5 g of ZnCl_2_ for 10 min at 105 °C and with a microwave power of 400 W to yield 87.4% esculetin (Scheme 3).

## 4. Synthesis and Pharmacological Evaluation of Esculetin Derivatives

**Scheme 4:**
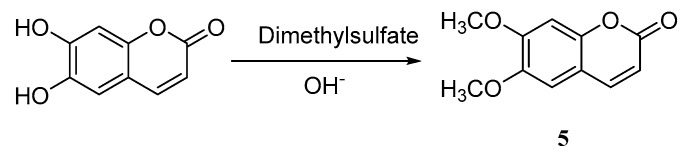
Synthesis of 6,7-dimethoxycoumarin.

Zhang et al. [40] reported the synthesis of 6,7-dimethoxycoumarin **5** by methylation from esculetin with an overall yield of 74.4% (Scheme 4). Zhao [41] utilized the same route to synthesize 6,7-dimethoxycoumarin, of which catalysts in the methylation progress of 6,7-dihydroxycoumarin were investigated and optimized. The author compared the catalytic effects in the methylation process with [BMIm][BF_4_], [BMIm]Cl, [BMIm]Br, and [BMIm][PF_6_] using phase-ransfer catalyst (PTC). The results indicated that the catalytic effect of *N*-dialkylimidazolium-based ionic liquid is better than PTC. Additionally, applying imidazolium ionic liquids as catalysts will not only increase the reaction yields, improve selectivity and the overall reaction under the low temperature, but the catalysts can also be reused.

The overexpression of tissue factor (TF) is one of the main causes of high levels of oxidized lipoproteins and blood cholesterol. In a biological study, 6,7-dimethoxycoumarin was shown to significantly reduce cholesterol and plasma lipids levels, which indirectly decreased the expression of TF in thrombus generation and exhibited potent antioxidant activities. Another research study [42] reported that 6,7-dimethoxycoumarin had potent inhibitory activity against clostridium histolyticum collagenase (ChC).

**Scheme 5:**
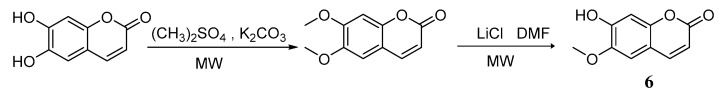
Synthesis route of 6-methoxycoumarin.

**Scheme 6:**
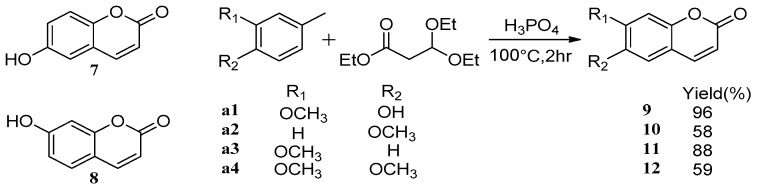
Synthesis route of esculetin analogues.

Compared with traditional methods [43,44], utilizing assisted microwave irradiation during scopoletin **6** synthesis could shorten the reaction time, increase reaction selectivity, and increase the yield from 46% using traditional heating and stirring to 73% [45] (Scheme 5). Adfa et al. [46] synthesized several scopoletin derivative analogues. According to biological screening, scopoletin showed the strongest termiticidal activity, followed by 6-methoxycoumarin **10**, 6-hydroxycoumarin **7**, and umbelliferone **8** (Scheme 6).

**Scheme 7:**
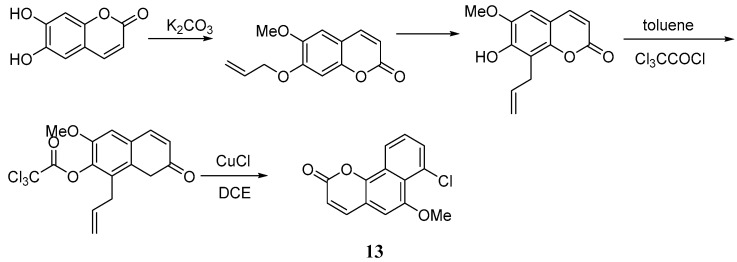
Synthesis of 6*H*-benzo[*d*]naphtha[1,2-*b*]pyran-6-one skeleton.

Bull et al. [47] put forward a new approach for the synthesis of the 6*H*-benzo[*d*]naphtha[1,2-*b*]pyran-6-one skeleton discovered in gilvocarcin. The percent yield of the coumarin **13** reached 84% after chromatography. The key of this synthetic route is the application of a new benzannulation strategy (the BHQ Reaction), whereby initial benzannulation and subsequent ortho-allylaryl trichloroacetate displacement reactions are transformed into highly regiospecific naphthalene derivatives, which enable the regiocontrolled synthesis of functionalized coumarin derivatives via an initial catalysed atom transfer radical cyclization (ATRC) reaction (Scheme 7).

**Scheme 8:**
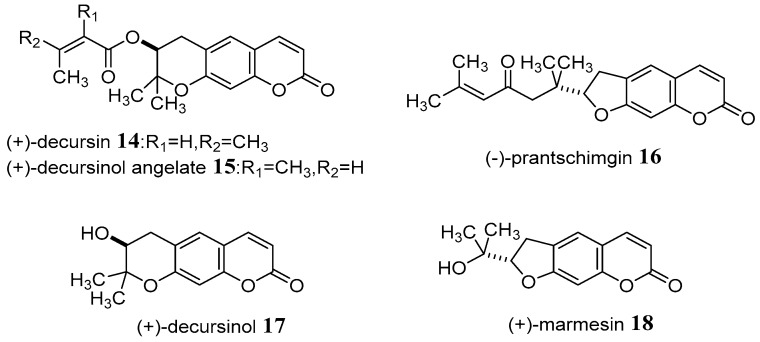
The structure of new coumarin derivatives.

Nemoto et al. [48] successively applied catalytic asymmetric epoxidation of an enone to enantioselective total synthesis a series of coumarin derivatives, including (+)-decursin **14** and related natural dihydropyranocoumarins (−)-prantschimgin **16**, (+)-decursinol **17**, as well as (+)-marmesin **18** for the first time. The new asymmetric catalyst composed of La(*O*-*i*-Pr)_3_, BINOL, and Ph_3_As = O in a 1:1:1 ratio was found to effectively promote catalytic asymmetric epoxidation of enone in 94% yield and 96% ee after recrystallization of the final product, providing optically pure epoxide (Scheme 8).

**Scheme 9:**
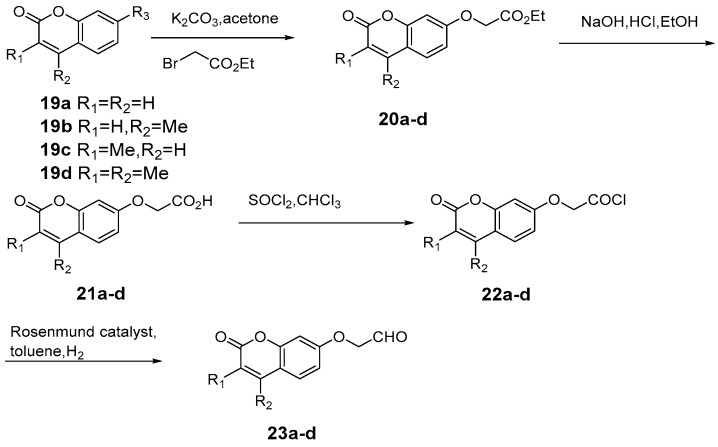
Synthesis route of 7-(2-oxoethoxy) coumarin.

**Scheme 10:**
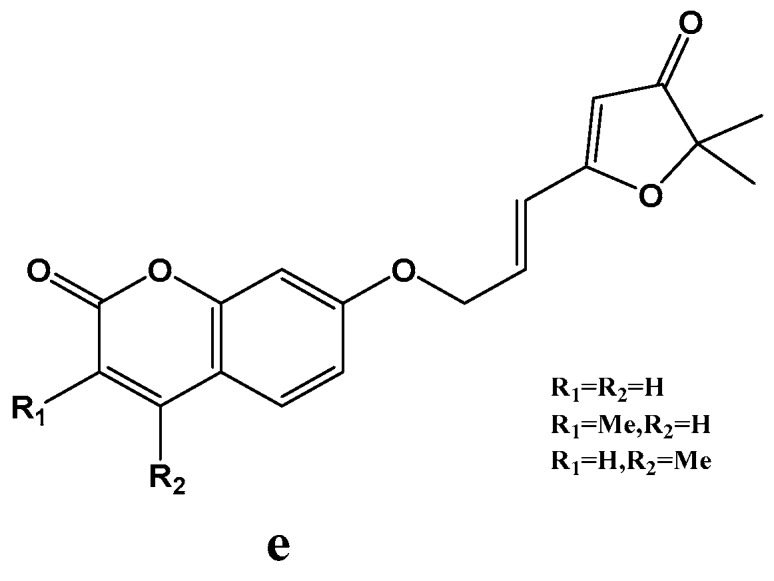
Structure of a series of natural geiparvarin analogues.

Chimichi et al. [49] developed a novel synthetic approach to coumarinyloxy aldehydes starting from simple hydroxyl coumarins, and converting to 7-(2-oxoethoxy) coumarins **23a**-**d** in much higher yields (Scheme 9). The author utilized this strategy in the synthesis of a new series of natural geiparvarin analogues **e**: classical aldolic condensation of 7-(2-oxoethoxy) coumarins with 3(2*H*)-furanones followed by an efficient dehydration. Moreover, this route has been applied to the synthesis of esculetin and other dihydroxycoumarins. Biologically, the new geiparvarin derivatives exhibited potent antitumour activity against several commercially available human cell lines. Geiparvarin and its synthetic analogues have been shown to act as inhibitors of twin monoamine oxidase isoforms, MAO-A and MAO-B (Scheme 10).

**Scheme 11:**
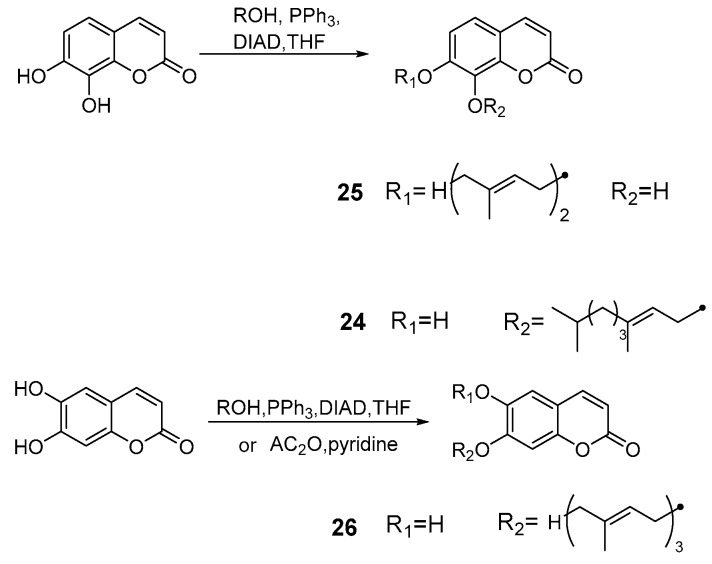
Mitsunobu monoalkylation of natural dihydroxycoumarins.

Cravotto et al. [50] developed the Mitsunobu monoalkylation of natural dihydroxycoumarins under high intensity sonochemical conditions. This method led to several compounds that showed high pharmacological activity or precursors thereof. The phytoestrogen ferujol **24** isolated from *Ferula jaeschkeana* [51] was synthesized under sonochemical conditions in good yield. In addition, for the first time, simple methylation of compound **25** afforded the anti-virus and anti-platelet aggregation agent collinine (from *Zanthoxylum schinifolium*). Another selective epoxidation of 7-farnesylesculetin **26** could also be developed as new squalene-hopene cyclase inhibitors [52] (Scheme 11).

**Scheme 12:**
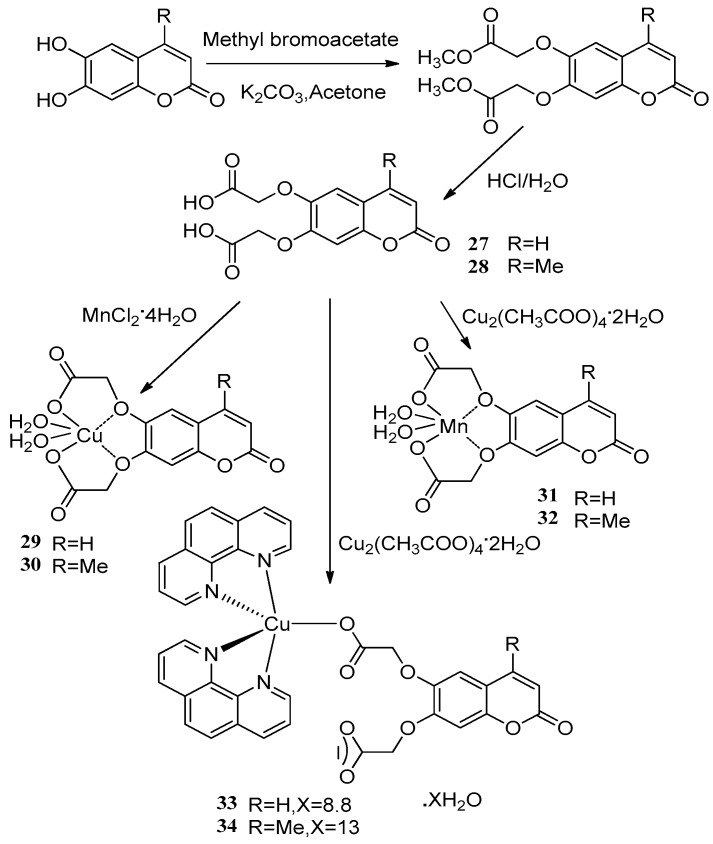
Synthesis route of two novel coumarin-based copper(II) and manganese(II) salts.

Creaven et al. [53] applied two novel coumarin-based ligands, coumarin-6,7-dioxyacetic acid **27** and 4-methylcoumarin-6,7-dioxyacetic acid **28** to react with copper(II) and manganese(II) salts affording the corresponding metal complexes. The metal complexes showed significant antimicrobial activity against some microbial species, including methicillin-resistant *Staphylococcus aureus* (MRSA), *Patonea agglumerans, E. coli* and *Candida albicans*. The metal-free ligands **27** and **28** were effective against all of the microbial species. Complexes **29**–**32** had no apparent activity compared with the metal-free ligands **27** and **28**, while the phenolic hydroxy adducts **33** and **34** inhibited MRSA (MIC_80_ = 12.1 μM), *E. coli* (MIC_80_ = 14.9 μM) and *Patonea agglumerans* (MIC_80_ = 12.6 μM). Complex **33** showed potent anti-*Candida* activity (MIC_80_ = 22 μM) versus the commercially available antifungal agent ketoconazole (MIC_80_ = 25 μ M) (Scheme 12).

**Figure 2:**
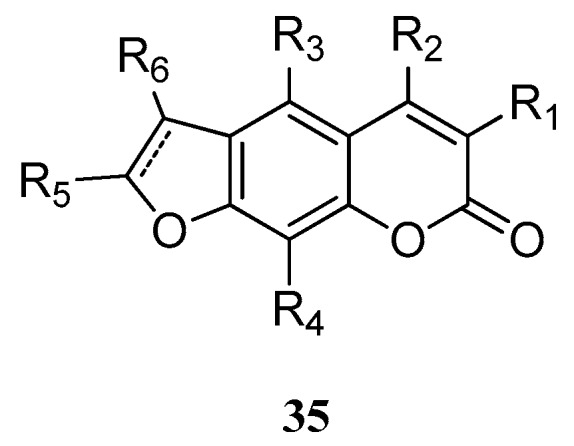
Structure of a series of furanocoumarin derivatives.

Hu et al. [54] reported a series of furanocoumarin derivatives **35** (Figure 2). These may be used as glucose transporter type 4 (GLUT4) membrane transfer agonists and GLUT4 protein expression agonists, and therefore can be used in the therapy of diabetes and its complications (wherein R_1_, R_2_, R_5_, and R_6_ = H, alkyl, alkenyl, or phenyl groups; R_3_ and R_4_ = H, alkyl, alkenyl, Ph, hydroxy, or alkoxy groups).

**Figure 3:**
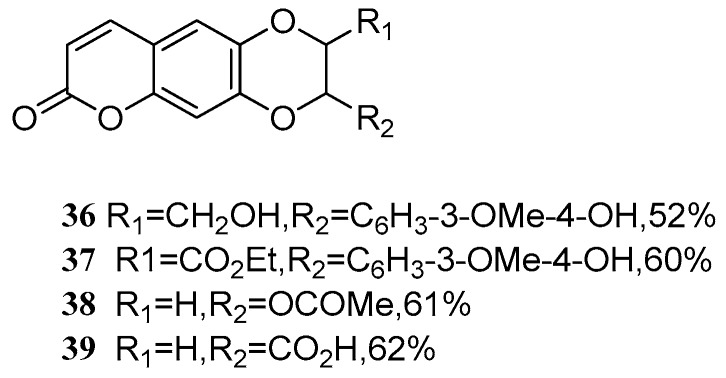
Structure of a series of novel coumarin olignans.

Bhardwaj et al. [55] investigated a catalyst for oxidative coupling of coumarin with propenyl phenols and alkene substrates. With the optimized catalyst in hand, a series of novel coumarin olignans **36**–**39** (Figure 3) were synthesized by dimerization through the C-O-C linkage.

**Scheme 13:**
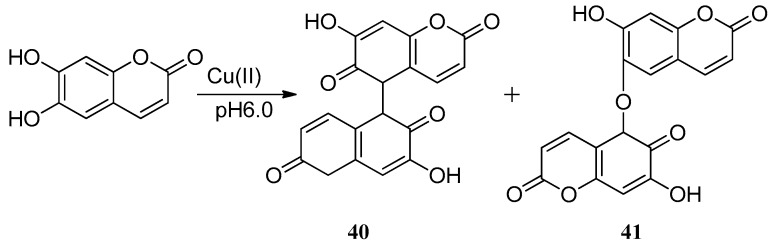
The method of forming dimers.

In aqueous phase, esculetin was also found to mobilize the Cu(II) ions accumulated in a Ca-polygalacturonate matrix (Ca-PGA). One molecule of esculetin can cause the reduction of one Cu(II) ion to Cu(I) ion with formation of oxidized semiquinonic radicals that form dimers **40** and **41** [56]. Castaldi et al. tested the ability of malic acid and esculetin to mobilize the Cu(II) ions. At pH 5.0 and 6.0, esculetin mobilizes approximately 12% and 25% of the Cu(II) accumulated. The research of Cu(II)-esculetin interactional activity also showed that with the formation of semiquinonic radicals, one molecule of esculetin reduces one Cu(II) ion, and the speed of Cu(II) reduction by esculetin was found to be faster in the presence of malic acid (Scheme 13).

**Figure 4:**
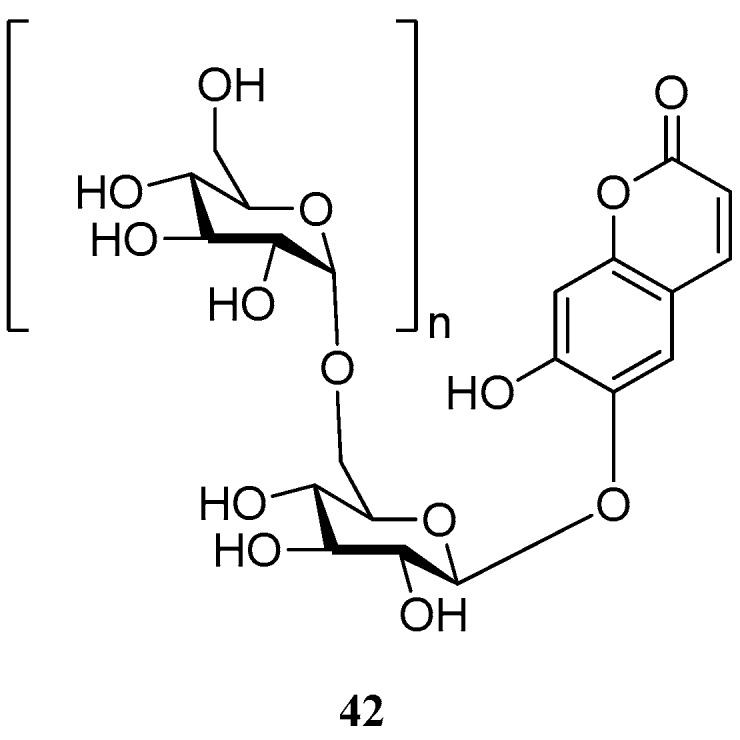
Structure of polysaccharide glycosides of esculetin.

Aurioll et al. [57] reported an invention which relates to polysaccharide glycosides of esculetin **42** (Figure 4). These new derivatives show a diversity of applications in cosmetic, nutrition and pharmaceutical compositions, such as treatment or prevention of oxidative stress, cancer, cardiovascular disease, bacterial infections, viral infections, fungal infections, UV-induced erythema, allergies, metabolism disorders, diabetes, obesity, hormonal disorders, bone disorders, pain, cerebrovascular disease, oral or dental diseases, inflammatory and immune disorders.

**Figure 5:**
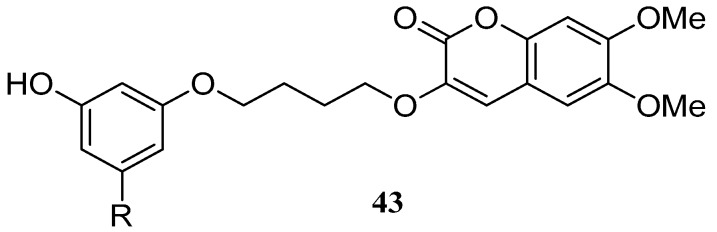
Structure of 6,7-dimethoxy-3-substituted coumarin.

Pisani et al. [58] synthesized a large group of substituted coumarins linked through a spacer to 3-hydroxy-*N*,*N*-dimethylanilino or 3-hydroxy-*N*,*N*,*N*-dialkyl benzaminium moieties. These coumarin derivatives were evaluated as acetylcholinesterase (AChE) and butyrylcholinesterase (BChE) inhibitors. The highest AChE inhibitory potency in the 3-hydroxy-*N,N*-dimethylanilino series was discovered with a 6,7-dimethoxy-3-substituted coumarin derivative **43** (Figure 5). Derivative **43** had an outstanding affinity (IC_50_ = 0.236 nm) and exhibited remarkable AChE/BChE selectivity (SI > 300,000).

## 5. Conclusions

Numerous studies have investigated the chemical synthesis and pharmacological activities of esculetin and its derivatives. Among the biological effects and molecular mechanisms of esculetin, antioxidant activity plays a crucial role associated with decreased levels of ROS/RNS, which is further potentially related to the anti-proliferative, anti-inflammatory, anti-phospholipid syndrome and other pharmacological activities. Most cirrhotic livers develop into liver cancer, and the common mechanism for development of hepatocellular carcinoma is chronic inflammation associated with severe oxidative stress, inducing liver cell nuclear shrinkage and fragmentation. Treatment with esculetin significantly reduced the over-expression tissue factor at mRNA levels in placenta. Thus, esculetin has also been investigated as a promising hypolipidemic agent and liver protectant. Phenolic 6,7-dihydroxy and 1-benzopyran-2-one scaffolds of esculetin provide convenient handles for esculetin derivative synthesis. Further clinical applications of esculetin and its synthetic derivatives may be developed based on well-understood pharmacological mechanisms both in vivo and in vitro. In future research, special attention will be focused on the potential for drug improvement, the research integrity of new esculetin derivatives and an in-depth mechanistic study in order to determine the clinically available agents.
